# Fine‐tuning the buzz: comparing visitation frequency and pollination effectiveness in plant–pollinator networks

**DOI:** 10.1111/nph.70758

**Published:** 2025-11-21

**Authors:** Lorena B. Valadão‐Mendes, Pamela C. Santana, André R. Rech, Vinícius L. G. Brito, Pietro K. Maruyama

**Affiliations:** ^1^ Programa de Pós‐graduação em Ecologia, Conservação e Manejo da Vida Silvestre Universidade Federal de Minas Gerais Belo Horizonte MG 31270‐901 Brazil; ^2^ Centro de Síntese Ecológica e Conservação, Departamento de Genética, Ecologia e Evolução, ICB Universidade Federal de Minas Gerais Belo Horizonte MG 31270‐901 Brazil; ^3^ Programa de Pós‐graduação em Biologia Animal Universidade Federal dos Vales do Jequitinhonha e Mucuri Diamantina MG 39100‐000 Brazil; ^4^ Departamento de Ciências e Linguagens, Instituto Federal de Educação Ciência e Tecnologia de Minas Gerais Bambuí MG 38900‐000 Brazil; ^5^ SpACE Group (Speciation, Adaptation and Coevolution), Department of Biology, Biodiversity and Evolution Unit Lund University Lund Skåne 223 62 Sweden; ^6^ Centro de Estudos Avançados em Funcionamento de Sistemas Ecológicos e Interações (CAFESIN) Universidade Federal dos Vales do Jequitinhonha e Mucuri UFVJM Diamantina MG 39100‐000 Brazil; ^7^ Instituto de Biologia Universidade Federal de Uberlândia Uberlândia MG 38405‐240 Brazil

**Keywords:** bees, buzz‐pollination, Campo Rupestre, *Chamaecrista*, pollen flowers, pollination

## Abstract

- Ecological network approaches have advanced our understanding of how species interactions influence community and evolutionary dynamics. However, a key limitation is that most network analyses rely solely on visitation data, often overlooking functional aspects of interactions. Here, we combined quantitative (visitation frequency) and qualitative (pollen removal and deposition) components to assess bee interactions with buzz‐pollinated flowers in the field.
- We recorded bee visitation to *Chamaecrista* (Fabaceae) flowers to represent the quantitative component and conducted single‐visit experiments to evaluate qualitative components related to male and female reproductive performances. Data were integrated into ecological networks to explore the structure of plant–pollinator interactions.
- Across 1838 interactions involving 10 plant species, flower‐buzzing bees were the most effective pollen depositors, while robbers removed large amounts of pollen but frequently damaged floral structures. Network analyses revealed that male performance components generated more specialised and modular networks than those based on visitation or female performance, highlighting functional differences among bee groups.
- While visitation networks offered partial insights, inclusion of pollination effectiveness metrics revealed the importance of specialised vibration behaviours in plant reproductive performance. We emphasise integration of both quantitative and qualitative data to better predict ecological and evolutionary dynamics in specialised pollination systems.

## Introduction

Biotic pollination, in which pollen transfer relies on animals, is an essential interaction for most flowering plants, affecting ecological and evolutionary processes in terrestrial ecosystems (Faegri & Van Der Pijl, 2013; Tong *et al*., 2023). In this context, interactions between plants and pollinators at the community scale have received many new insights with the use of ecological network approaches (Vázquez *et al*., 2005; Bascompte & Jordano, 2007; Magrach *et al*., 2021). Usually, pollination networks are built using quantitative data on observed visitation and/or indirect evidence, such as pollen load on the body of a pollinator (Vázquez *et al*., 2005; Vizentin‐Bugoni *et al*., 2018; Souza *et al*., 2021; Carneiro *et al*., 2024). However, quantifying plant–pollination interactions solely based on the number of recorded visits to flowers from individual plants may poorly represent pollination effectiveness (i.e. the joint contribution of both quantitative and qualitative components; Ballantyne *et al*., 2015; Schupp *et al*., 2017; González‐Castro *et al*., 2022). Since most pollination networks rely on visitation frequency as the measure/currency for interaction strength, it raises the question: how accurately is the pollinator role depicted in ecological networks (Vázquez *et al*., 2005; King *et al*., 2013; Ballantyne *et al*., 2015; González‐Castro *et al*., 2022)? Furthermore, the effectiveness of pollination can be assessed from both paternal (seeds sired) and maternal outcomes, considering that pollinators differently influence plants' male performance through pollen removal and female performance through pollen deposition (Minnaar *et al*., 2019; Bergamo *et al*., 2023). Therefore, a deeper understanding of pollinator roles in interaction networks requires the addition of more informative currencies through the weighting of visitations by measures of pollination effectiveness (King *et al*., 2013; Blüthgen & Staab, 2024).

The investigation of each pollinator species' effectiveness in plant–pollinator interactions may allow for disentangling key species for ecosystem functioning (Schupp, 1993; Schupp *et al*., 2017). Highly effective species in pollination interactions will be the ones that contribute significantly to reproductive success, most directly affecting the population dynamics of their partners (Schupp, 1993; Santiago‐Hernández *et al*., 2019; Gómez *et al*., 2022). Consequently, they may impose stronger selective pressures on traits mediating the interactions, driving their phenotypic evolution (Valverde *et al*., 2019; Valadão‐Mendes *et al*., 2022). For pollination interactions, the quality component of interaction is directly connected to pollen transfer, through removal from anthers and deposition on stigmas, representing male and female reproductive performances, respectively (Minnaar & Anderson, 2019; Opedal *et al*., 2023). Male reproductive performance refers to pollen export from flowers, mediated in most cases by pollinators, such as bees (Thomson, 1986; Opedal *et al*., 2023). By contrast, female reproductive performance relates to the reception of pollen on stigmatic surfaces and the subsequent fruit and seed sets (Fontúrbel *et al*., 2017; Schupp *et al*., 2017; Gómez *et al*., 2022). Both male and female performances determine the final reproductive success of a plant (Ne'eman *et al*., 2010; Cortés‐Rivas *et al*., 2023). Interestingly, each component may impose distinct selective pressures on flower traits involved in the interaction with pollinators (Delph & Ashman, 2006; Santana *et al*., 2025). Therefore, decomposing male and female reproductive performances may provide a more nuanced understanding of different evolutionary processes mediated by pollination (Santiago‐Hernández *et al*., 2019; Gómez *et al*., 2022).

Previous studies have incorporated the qualitative components of pollination within networks, considering data on flower handling (e.g. Mesquita‐Neto *et al*., 2018), pollen deposition on stigmas (King *et al*., 2013; Ballantyne *et al*., 2015) and subsequent measures such as fruit and seed sets (Santiago‐Hernández *et al*., 2019). These studies demonstrated idiosyncrasies stemming from the type of data used to build interaction networks that lead to changes in network structures (Souza *et al*., 2021). Other studies at the species level have demonstrated the importance of considering variables such as pollen deposition to determine the pollination effectiveness of different floral visitors (e.g. Barrios *et al*., 2016). Nevertheless, all these studies focused on the female reproductive performance of plants, even though the male reproductive performance may operate in substantially different ways from the female one (Moore & Pannell, 2011; Minnaar & Anderson, 2019). For instance, some pollinators may be better at removing pollen from the anthers than at delivering it onto stigmas because pollen transport faces different barriers, such as pollen theft (Mesquita‐Neto *et al*., 2018) and bee grooming behaviour (Koch *et al*., 2017; Tong & Huang, 2018; Marcelo *et al*., 2022). Furthermore, plant female reproductive performance as a function of flower visitation may saturate differentially than male performance (Janzen, 1977; Gong & Huang, 2014; Bergamo *et al*., 2023). Therefore, an integrated comprehension of the effects of pollination interaction on the ecology and evolutionary dynamics playing at the community scale needs to account for both the male and the female reproductive performances (Karron *et al*., 2006; Bergamo *et al*., 2023).

Buzz pollination is a specialised interaction involving plants with distinct floral traits and a unique behaviour displayed by bees when collecting pollen (Vallejo‐Marin & Russell, 2024). The floral traits exhibited by buzz‐pollinated plants are characterised by (1) pollen released through tiny pores or apical slits (i.e. poricidal anthers), (2) functional specialisation of stamens (i.e. heteranthery) and (3) pollen grains as the main floral resources (i.e. pollen‐only flowers) (Vogel, 1978; Buchmann, 1983; Luo *et al*., 2008; Vallejo‐Marín *et al*., 2010). These flowers are often visited by insects, mostly female bees (but not honeybees) and sometimes syrphid flies (e.g. *Copestylum mexicana*), capable of performing vibrations to extract pollen grains, used to feed their larvae (Buchmann & Hurley, 1978; Buchmann, 1983; Vallejo‐Marín, 2022). Since pollen grains are enclosed in specialised poricidal anthers, it facilitates the measurement of pollen removal by pollinators (Papaj *et al*., 2017; Brito *et al*., 2021), allowing simultaneous investigation of both male and female reproductive performance components (Barrios *et al*., 2016). Therefore, the interaction network built between plants with pollen flowers and potential pollinators is an excellent model system to separately investigate the male and female performance that make up the quality component of the effectiveness in the construction of pollination networks.

In this study, we took advantage of a natural buzz pollination system to evaluate plant–pollinator interactions and the relative importance of different species to specific fitness components by comparing their resulting plant–pollinator networks. We used buzz‐pollinated flowers from the highly diverse genus *Chamaecrista* (Fabaceae), known for considerable variation in floral traits paralleled by specialised associations with pollen‐harvesting bee pollinators (Thorp & Estes, 1975; Gottsberger & Silberbauer‐Gottsberger, 1988; Wolfe & Estes, 1992; Tucker, 1996; Nogueira *et al*., 2018). More specifically, we address the following questions: (1) how do pollen removal from anthers (male performance) and pollen deposition onto stigmas (female performance) vary among different floral visitors? (2) does bee species contribute differently to each component of the plant reproductive performance (male and female)? (3) do networks built only with quantitative components (i.e. visitation rates) differ from networks built including qualitative components (i.e. male and female performances)? (4) do node‐level metrics change according to which component was used to build the networks? We hypothesised a lack of association between the removal and deposition of pollen grains, meaning that each interaction would have different genderwise reproductive performances. By incorporating male and female performances mediated by floral visitation, we expect to find more specialised networks when including the quality component, as only some of the visits translate into effective pollination. Our findings and the procedures illustrated here contribute to a more precise description of the community patterns associated with pollination interactions, and consequently, the dynamics these interactions mediate.

## Materials and Methods

### Study area and plant species

**Fig. 1:**
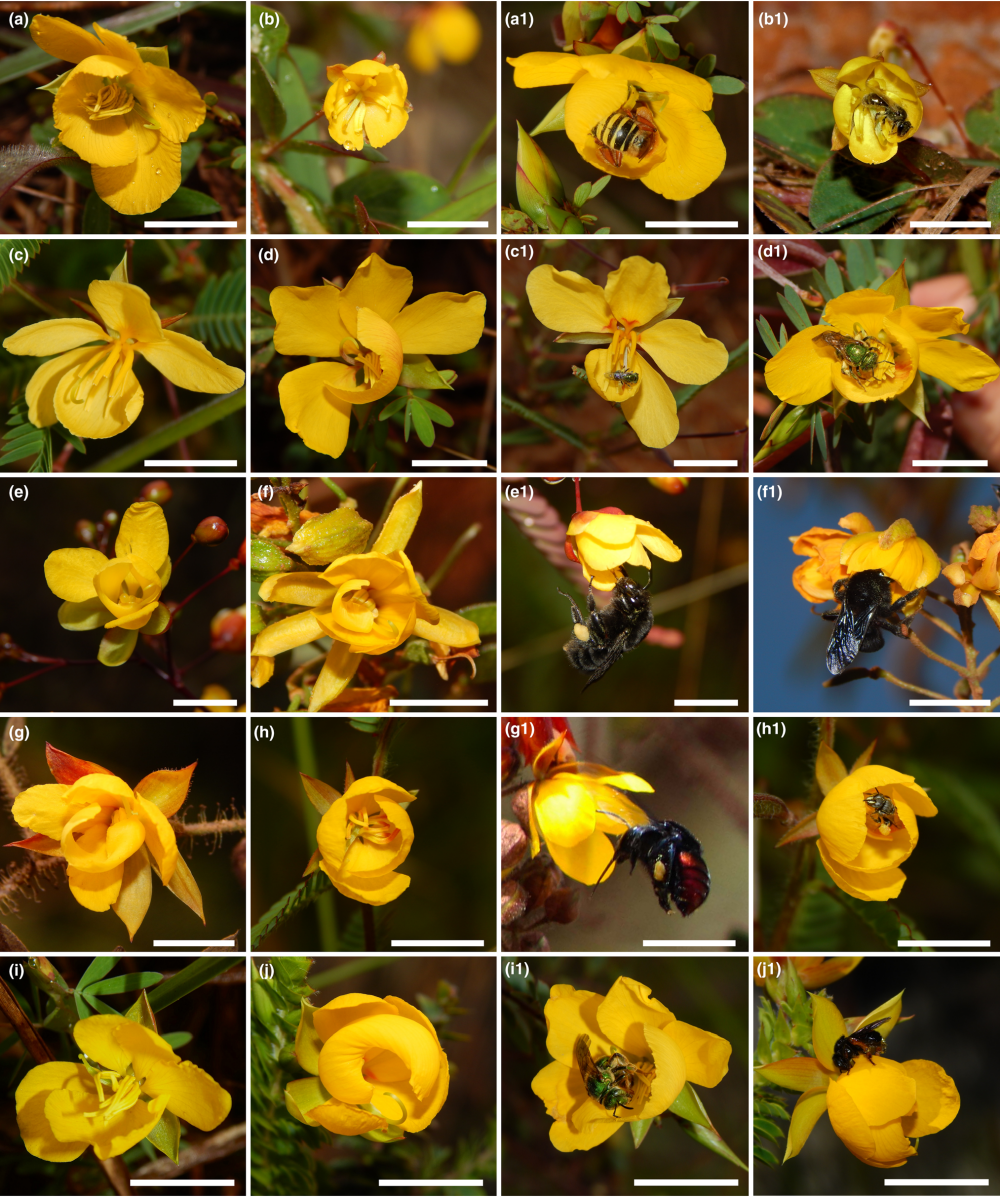
Flowers and floral visitors of *Chamaecrista*. (a, a1) *Chamaecrista ramosa* (Vogel) H.S. Irwin & Barneby and its floral visitor *Melipona quinquefasciata*. (b, b1) *Chamaecrista rotundifolia* (Pers.) Greene and its floral visitor *Dialictus* sp. 13. (c, c1) *Chamaecrista flexuosa* (L.) Greene and its floral visitor *Augochlora* sp. 05. (d, d1) *Chamaecrista ramosa* (Vogel) H.S. Irwin & Barneby var. 1 and its floral visitor *Augochloropsis* sp. 50. (e, e1) *Chamaecrista debilis* (Vogel) H.S. Irwin & Barneby and its floral visitor *Bombus pauloensis*. (f, f1) *Chamaecrista hedysaroides* (Vogel) H.S. Irwin & *Barneby* and its floral visitor *Bombus morio*. (g, g1) *Chamaecrista bracteolata* (Vogel) H.S. Irwin & Barneby and its floral visitor *Eufriesea nigrohirta*. (h, h1) *Chamaecrista nictitans* (L.) Moench and its floral visitor *Paratrigona subnuda*. (i, i1) *Chamaecrista desvauxii* (Collad.) Killip and its floral visitor *Augochloropsis* sp. 50. (j, j1) *Chamaecrista distichoclada* (Benth.) H.S. Irwin & Barneby and its floral visitor *Trigona spinipes*. Bars, 1 cm.

We conducted the study in the Brazilian *Campos Rupestres*, a rupestrian grassland ecosystem with a mosaic of open vegetation where herb and shrub species predominate between stretches of rocky outcrops (Silveira *et al*., 2016). The *Campos Rupestres* are recognised by their high biodiversity and endemism of plants and pollinators (Silveira *et al*., 2016; Monteiro *et al*., 2021), comprising a great richness of coflowering plant species with poricidal anthers throughout the year. We conducted the data collection in the surroundings of the Federal University of the Vales do Jequitinhonha e Mucuri (UFJVM – 18°11′S, 43°34′W), located in Diamantina municipality, Minas Gerais, Brazil, from February 2021 to April 2022. The study area harbours many buzz‐pollinated species from Melastomataceae, Solanaceae and Fabaceae families, the latter including species from *Chamaecrista* and *Senna* genera. Among these, *Chamaecrista* (L.) Moench represented the most abundant buzz‐pollinated plant group in the area and is generally widely distributed across *Campos Rupestres* vegetation. We selected the 10 *Chamaecrista* species with the highest floral abundance for detailed observation (Fig. 1), but up to 10 other buzz‐pollinated species have been identified in the area during the study period. *Chamaecrista* is a mostly shrub‐like genus with asymmetric corolla composed of five petals (Fig. 1; Irwin & Barneby, 1982). The reproductive structures usually display enantiostyly and dimorphic anthers (Irwin & Barneby, 1982). Flowers offer only pollen as a floral resource (called pollen flowers), bear anthers with poricidal dehiscence and pollen grains are released only when bees vibrate the anthers (e.g. buzz pollination). Released pollen grains are often directed onto a bee's body by a ricochet mechanism involving a modified petal known as *cucculus* (Westerkamp, 2004; Amorin *et al*., 2017; Monteiro *et al*., 2025). Anthesis is usually diurnal and flowers last just 1 d. The fruit is linear, dry and of legume type (Irwin & Barneby, 1982).

In the study area, we selected and georeferenced *c*. 10 individuals (mean ± SD = 10 ± 3) from each of the 10 *Chamaecrista* species. We selected the species based on the intensity of flowering during the data collection as well as to represent the wide variation of floral morphology and size (ranging from 0.8 to 4.5 cm in diameter, see Fig. 1). We recorded three measures from the pollination interaction during the study period: visitation frequency, pollen removal and pollen deposition detailed below. Plant specimens were deposited at the HUFABC Herbarium at the Federal University of ABC (São Bernardo do Campo, São Paulo, Brazil) and identified with the assistance of a taxonomic specialist (see the Acknowledgements section).

### Currencies used to estimate the interaction outcome and weight the networks

#### Bee visitation frequency: quantitative component

**Fig. 2:**
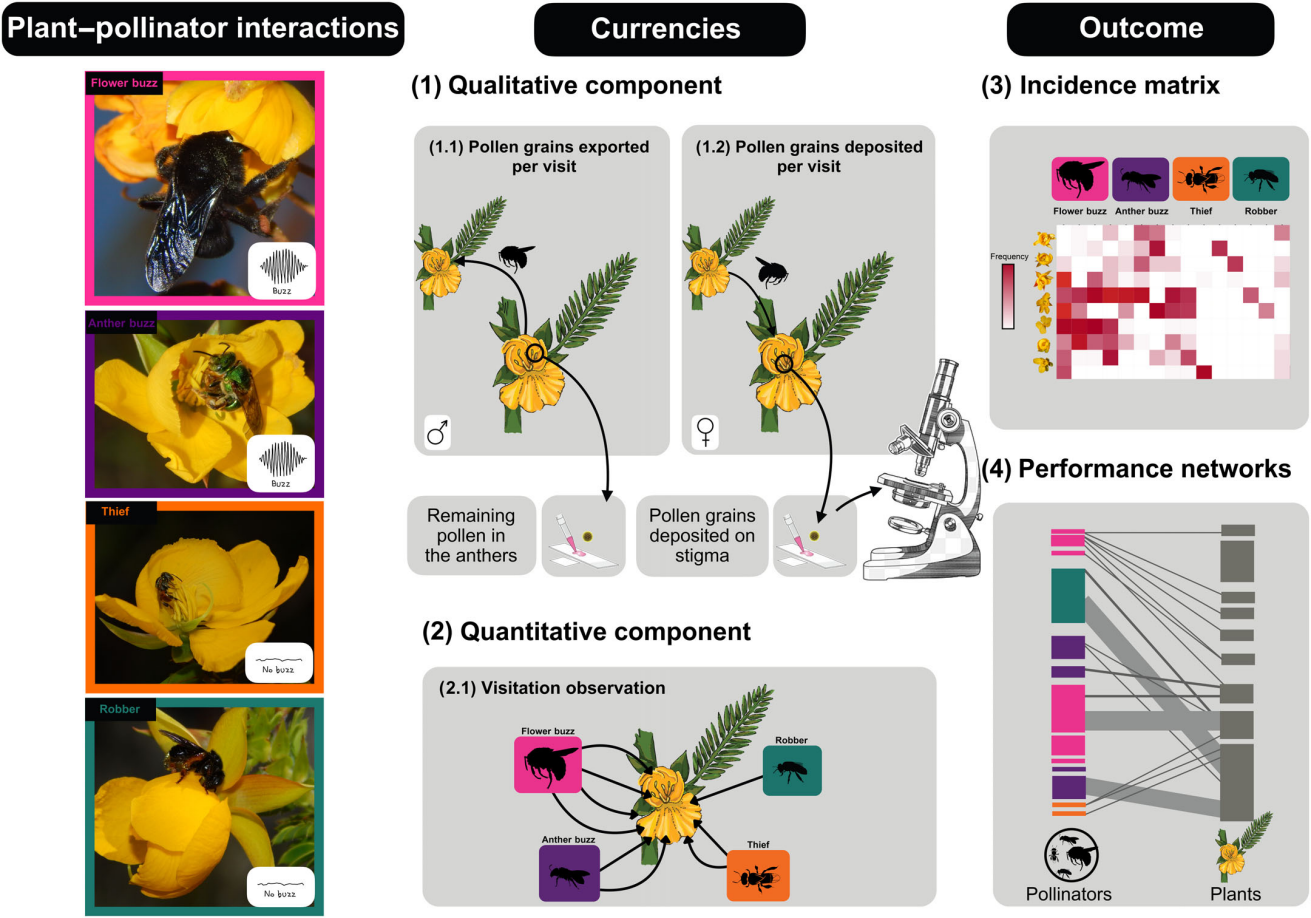
Illustrative workflow of our procedures: Left panel: interactions between *Chamaecrista* flowers and bees, with illustrative examples from each of the four functional groups. These interactions were then used to build interaction networks, represented by (1) qualitative component assessed through single‐visit experiments; (2) quantitative component representing the frequency of pairwise interactions; which are then (3) combined into a matrix and finally, the data from (1) and (3) are combined into (4) performance networks for male and female components. Pollinator images were obtained from https://phylopic.org/.

We sampled bee visitation frequency during 70 sunny days in 2021 (from March to December) and 21 sunny days in 2022 (January to March). We performed focal observations on *c*. 10 flowering individuals (mean ± SD = 10 ± 3) per species to estimate flower visitation data (Fig. 2). We observed each individual for 30 min and recorded all flower visitors and their behaviour from 06:00 to 14:00 h, the peak activity period for the bees in the area. The observation period was defined to encompass both the peak of bee visitation and the longest duration of floral longevity after flower opening for the studied species, thereby ensuring representative coverage of interactions throughout the day. Every 30 min, we changed to another individual of the same plant species throughout the day. For each visit, we noted (1) bee species, (2) bee behaviour during pollen collection (i.e. buzzing or no buzzing) and (3) if they touched the reproductive structures. We performed a total of 556 h of observation, resulting in *c*. 56 ± 9 (mean ± SD) h per plant species. Bee specimens were collected and taken back to the laboratory for identification with the assistance of a taxonomic specialist (see the Acknowledgements section) and deposited at the Entomological Reference Collection of the Federal University of Minas Gerais, UFMG, Belo Horizonte, Brazil.

#### Single visit experiments: qualitative component

##### Estimation of the male performance

We conducted single‐visit experiments for each species from October 2021 to April 2022, between 07:00 and 11:00 h, to estimate pollen removal and deposition *efficacy per visit* during pollination interaction. We used from 5 to 10 flowering individuals per species, depending on flower availability. To conduct the experiment, we bagged flower buds of all plant species each afternoon. The following day, once the flowers opened, the bags were removed and exposed to floral visitors. We collected flowers as soon as they received their first bee visit. Subsequently, we transferred each flower to 50 ml Falcon tubes and recorded the visitor and plant identities. We performed the single‐visit experiments every day until there were no more bagged flowers or flower buds left to sample. A total of 908 flowers (mean ± SD = 67 ± 23) were sampled.

We estimated the quantity of pollen grains removed from the anthers during each bee visit as a proxy of male performance (Fig. 2). After a visit, (1) we took the flowers to the laboratory, removed the anthers and transferred them to 1.5 ml Eppendorf tubes; (2) we macerated the anthers with a plastic rod and added 240 μl of acetic carmine and glycerine (3 : 1) solution; (3) we stirred the tubes using the vortex for 60 s, transferred 10 μl of the solution to a Neubauer chamber and (4) we counted the pollen grains using light microscopy (×100 or ×400). Additionally, we collected unvisited bagged flowers to use as a reference (*n* = 10, per species) for the total number of pollen grains, which were subjected to the same procedure detailed previously. Finally, we subtracted the number of pollen grains remaining in the single‐visit samples from the average number of pollen grains in the reference samples to estimate the amount of pollen removed (Barbosa *et al*., 2023; Trevizan *et al*., 2023). To account for potential errors with counting pollen grains on the Neubauer camera, we followed the protocol published by Barbosa *et al*. (2023), where we used six replicates to estimate the average total number of pollen grains per flower. In this procedure, the coefficient of variation among replicates from the same flower was calculated to assess the consistency of the estimates and identify outlier values.

##### Estimation of the female performance

We quantified the number of pollen grains deposited on stigmas during each bee visit as a proxy of the female performance component (Fig. 2). We used the same flowers from the single‐visit experiments detailed above. After removing the anthers to estimate pollen removal, we (5) removed the stigma from the flower, arranged it on glass microscope slides with fuchsin jelly and covered it with coverslips; (6) we counted the number of conspecific pollen grains under light microscopy (×100 or ×400). As a control for self‐pollination, we counted the pollen grains present on the stigma from the unvisited flowers (*n* = 10 per species). We estimated a proxy for female performance by subtracting the average number of pollen grains in control flowers from the total number of pollen grains deposited in the single‐visit samples.

### Characterisation of visitor functional group

Bees were characterised into functional roles according to their behaviours when manipulating the flowers and collecting pollen (Mesquita‐Neto *et al*., 2018). The four groups are (1) *thief* – bees that collect pollen with their mouthparts from the apical pore and/or collect pollen by squeezing the anthers with their mouthparts for pollen removal and/or glean residual pollen from floral parts such as the petals after pollen extraction by vibrating bees. Thief bees generally do not cause floral damage. This group included four species (*Apis mellifera* Linnaeus, *Paratetrapedia lineata* (Spinola, 1853), *Paratrigona subnuda* Moure, 1947 and *Tetragonisca angustula* (Latreille, 1811)); (2) *robber* – bees that collect pollen cutting and perforating anthers and/or petals of flowers, always causing floral damage, which included two species (*Trigona spinipes* (Fabricius, 1793) and *Trigona braueri* Friese, 1900); (3) *anther buzzing* – bees that collect pollen by vibrating single anthers or a subset of available anthers. These bees alter their position within the flower between vibrating actions and included 17 species (*Augochlora* sp. 05, *Augochloropsis* sp. 49, *Augochloropsis* sp. 50, *Augochloropsis* sp. 51, *Augochloropsis* sp. 52, *Augochloropsis* sp. 53, *Caenohalictus* sp. 01, *Centris* (Trachina) *fuscata* Lepeletier, 1841, *Centris* (Hemisiella) *tarsata* Smith, 1874, *Centris* (Hemisiella) *trigonoides* Lepeletier, 1841, *Ceratina* (Ceratinula) *minima* Friese, 1908, *Dialictus* sp. 13, *Exomalopsis* (Exomalopsis) *fulvofasciata* Smith, 1879, *Melipona* (Melikerria) *quinquefasciata* Lepeletier, 1836, *Pseudaugochlora flammula*, *Pseudaugochlora graminea* Almeida, 2008 and *Thectochlora alaris* (Vachal, 1904)); (4) *Flower buzzing* – bees that collect pollen by vibrating all the anthers (or almost all) simultaneously and applying one or more vibration pulses. These bees generally do not change their position during a visit to the flower and included six species (*Bombus* (Fervidobombus) *morio* (Swederus, 1787), *Bombus* (Fervidobombus) *pauloensis* Friese, 1913, *Xylocopa* (Dasyxylocopa) *fortuita* Melo, 2017, *Xylocopa* (Neoxylocopa) *hirsutissima* Maidl, 1912, *Xylocopa* (Schonnherria) *subcyanea* Pérez, 1901 and *Xylocopa truxali* Hurd & Moure, 1963).

### Plant–pollinator network characterisation

To compare the effect of the different currencies on the network structure, we built five different bipartite networks, according to different combinations of our data: (1) *visitation network* (VN) – using the frequency of interaction between visitor groups and plant species; (2) *pollen deposition network* (DN) – considering the average number of conspecific pollen deposited on stigma during a single visit by bee species, for each pairwise bee – flower combination; (3) *female performance network* (FPN) – combining data of visitation frequency and pollen deposition for each pairwise interaction; (4) *pollen release network* (RN) – the average amount of pollen released during a single visit by bee species of each plant species and (5) *male performance network* (MPN) – combining data on visitation frequency and pollen removal by visitors of each plant species. After that, we calculated network metrics characterising structural properties of these networks (to be described later). The networks were constructed using the frequency of interactions between individual bee species and plant species. For interpretation and visualisation, bee species were subsequently categorised into four functional groups previously described, and node colours in figures reflect these groups.

### Data analyses

To analyse the effect of the visitor functional group on pollen deposition, we used a zero‐inflated model combined with a truncated negative binomial distribution of the response variable. The fixed factor was the treatment (functional group – categorical), and *Chamaecrista* species (categorical) was treated as a random intercept. To analyse the effect of the visitor functional group on visitation frequency and pollen export, we used a hurdle model in which the response variable followed a truncated negative binomial distribution. We used functional group (categorical) as a fixed effect and *Chamaecrista* species (categorical) as a random factor. Because our data showed heteroscedasticity, we modelled the variance according to an interaction between functional group and bee species, in addition to *Chamaecrista* species. We compared these models to a null model using the Akaike information criterion (AIC).

To characterise the structural properties of the different networks, we calculated the following metrics: (1) *nestedness* (NODF) – quantifies a nested pattern in which less connected species (specialists) interact with proper subsets of partners of the more connected species (generalists) (Almeida‐Neto & Ulrich, 2011); (2) *modularity* (*Q*_w_) – measures the extent to which interactions occur more frequently within defined subsets/groups of species (modules) in comparison to interactions between modules (Beckett *et al*., 2014) and (3) *specialisation* (*H*_2_′) – which quantifies the degree to which species deviate their use of resources from what is expected based on the availability of these resources, approximated by the total number of interactions/marginal totals (Blüthgen *et al*., 2006). The significance of *NODF*, *modularity* (*Q*_w_) and *specialisation* (*H*_2_′) was assessed by comparing the observed values to those obtained by the *r2dtable* null model (bipartite R package). We ran the null model 1000 times and evaluated the significance based on 95% confidence intervals of randomised values. We also calculated generality (Bersier *et al*., 2002) for each trophic level and species‐level metrics for strength and species‐level specialisation (*d*′) within networks. We conducted a *t*‐test to compare species strength and specialisation *d*′ metrics between the VN, FPN and MPN networks. Besides, we performed *cor.test* analyses to assess relationships among different network metrics derived from bee to plant interactions, focusing on VN, pollen DN and pollen RN. These analyses were conducted in three distinct contexts: species strength, specialisation (*d*′) and modularity (*c* and *z* values). Notably, we did not perform correlation tests for effectiveness networks (which combine visitation data with female and male effectiveness measures), as these composite metrics would inherently generate strong positive correlations due to their shared underlying components. Finally, to analyse the role of each species in the modular structure of the network, we calculated *z* values and *c* values (Olesen *et al*., 2007). The *z* value represents within‐module connectivity, whereas the *c* value captures connectivity between modules. The species were categorised as follows: (1) *peripheral species* (*z* ≤ 2.5 and *c* ≤ 0.62), characterised by having few connections within their own module and almost none with other modules; (2) *module hubs* (*z* > 2.5 and *c* ≤ 0.62), species that play a key role in maintaining the integrity of their respective modules; (3) *connectors* (*z* ≤ 2.5 and *c* > 0.62), species essential for linking different modules and ensuring coherence within their own module and (4) *network hubs* (*z* > 2.5 and *c* > 0.62), species critical for the cohesion of the entire network as well as their respective modules (Olesen *et al*., 2007).

All analyses were performed in R (R v4.4; R Development Core Team, 2023), using the main packages lme4 (Bates *et al*., 2015), glmmtmb (Brooks *et al*., 2017), dharma (Hartig, 2016), bbmle (Bolker & R Development Core Team, 2023), performance (Lüdecke *et al*., 2021), ggeffects (Lüdecke, 2018), mass (Ripley, 2015), pscl (Jackman, 2015), aer (Kleiber *et al*., 2020) and bipartite (Dormann *et al*., 2008). The complete list of packages, together with the code and data, is available at Github (https://github.com/SantanaPC/NetworkCurrencies).

## Results

### Variation in plant–pollinator interaction outcome

**Fig. 3:**
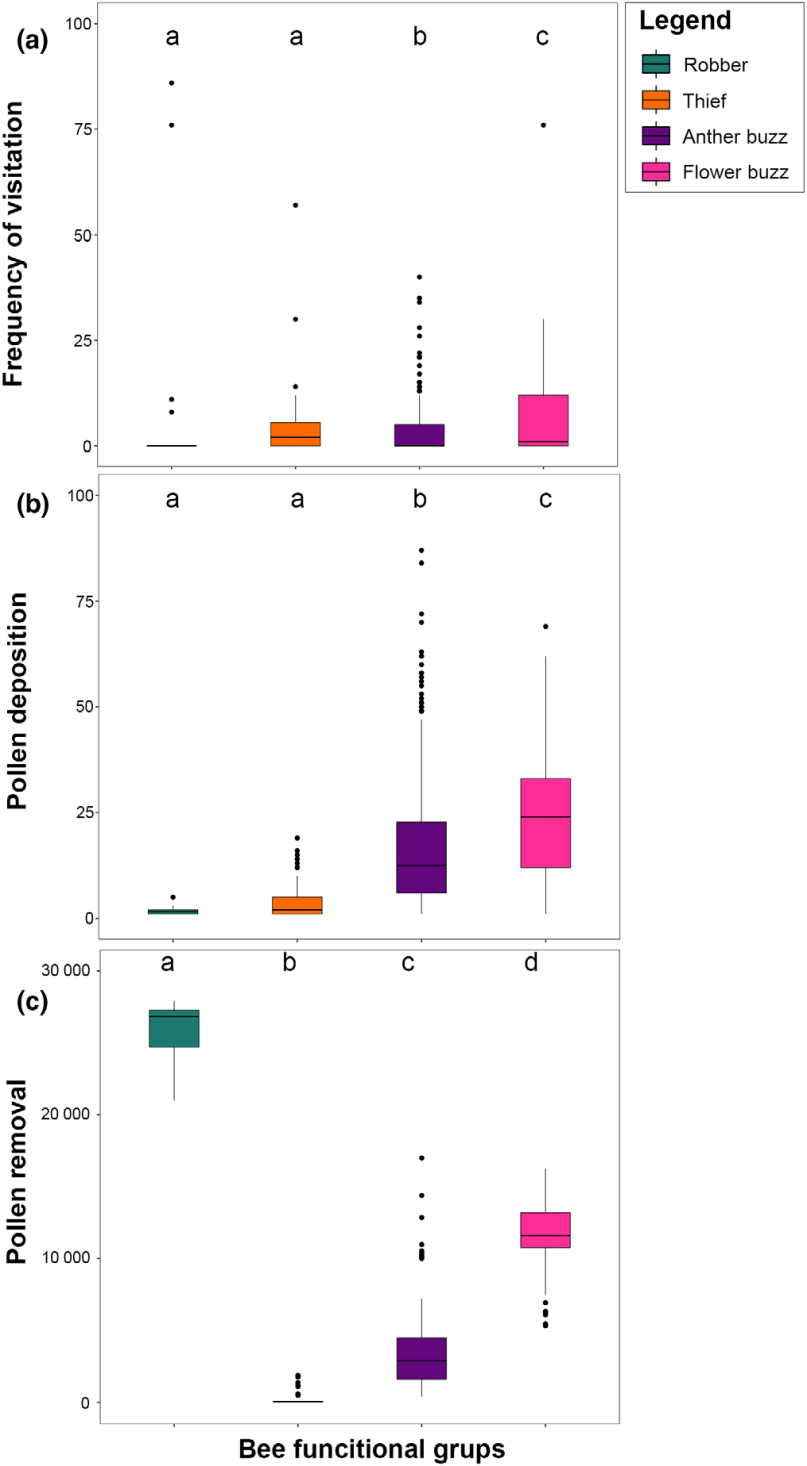
Box plots showing the bee functional groups considering: (a) the visitation frequency of each group, (b) the quantity of single‐visit pollen deposition of plant species, (c) the quantity of single‐visit pollen removal of plant species. Bars are coloured according to the bee's functional group, as indicated in the legend of the plot. Box plots show the median (horizontal line), boxes represent the interquartile range (25^th^–75^th^ percentiles), whiskers extend to 1.5× the interquartile range, and points beyond the whiskers indicate outliers. Different letters indicate significant differences (*P* ≤ 0.01) values according to Pairwise *t*‐tests.

We recorded a total of 1838 interactions between 33 bee species and 10 *Chamaecrista* species. *Bombus pauloensis* was the most frequent bee (25% of the total bee visitation); however, the number of visits varied between plant species. When focusing on each bee functional group separately, the most frequent flower manipulation behaviour was *flower buzzing* (Fig. 3a). More specifically, *flower‐buzzing* and *anther‐buzzing* functional groups corresponded to 79% of the total visitation frequency. The functional groups presented different patterns of pollen removal and deposition for *Chamaecrista* species (Fig. 3b,c). Bees categorised as *flower buzzing* deposited the highest quantities of pollen on the stigmas (24.4 ± 14.8 pollen grains per visit; mean and SD, respectively; Fig. 3b), while bees categorised as *thieves* and *robbers* deposited the smallest quantity of pollen grains (3.6 ± 3.3). On the other hand, for pollen removal, *robber* bees were the group that removed the highest amount of pollen from the anthers (25 671.2 ± 2418.3), whereas *thief* bees removed the smallest quantities (141.22 ± 320.4) (Fig. 3c).

**Fig. 4:**
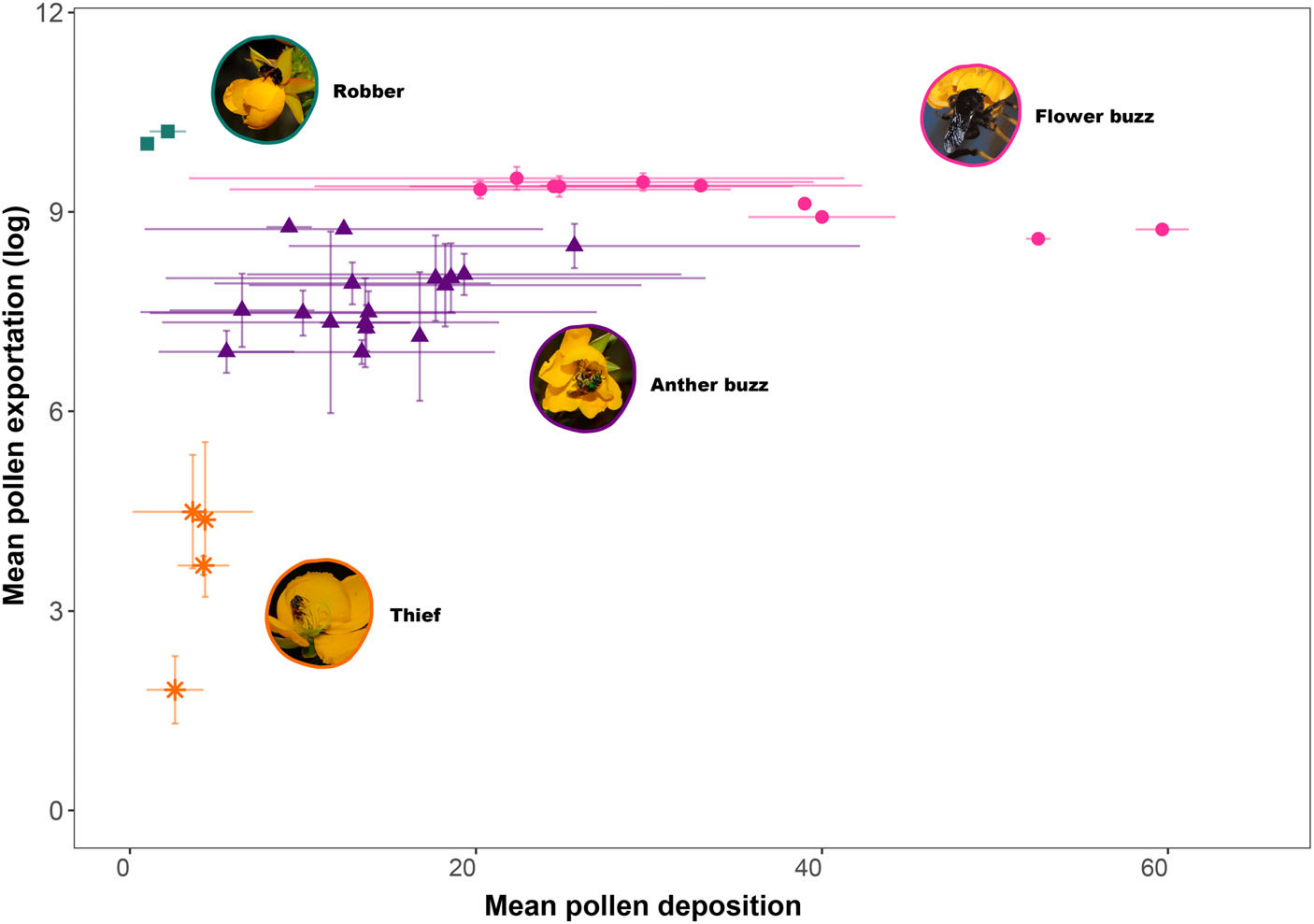
The effectiveness landscape representation considering the male and female performance contributed by bee visitors to *Chamaecrista* species. Lines indicate SD. Points are coloured according to the bee's functional group, as indicated in the legend.

Species within the same functional groups clustered together and exhibited similar efficacies in mediating both female and male plant performances (Fig. 4). Species belonging to the *robber* group showed higher pollen removal efficacy per visit to *Chamaecrista* species. Species in this functional group cut the anthers and removed nearly all the pollen. Bees in the *theft* functional group mediated lower male performance. However, when assessing female performance, we observed that species in the *flower‐buzzing* group were the most effective ones, followed by *anther buzzing*. Conversely, *robbers* and *thieves* were the groups with the least efficacy in depositing pollen.

### Variation in plant–pollinator network structural properties using different currencies

**Fig. 5:**
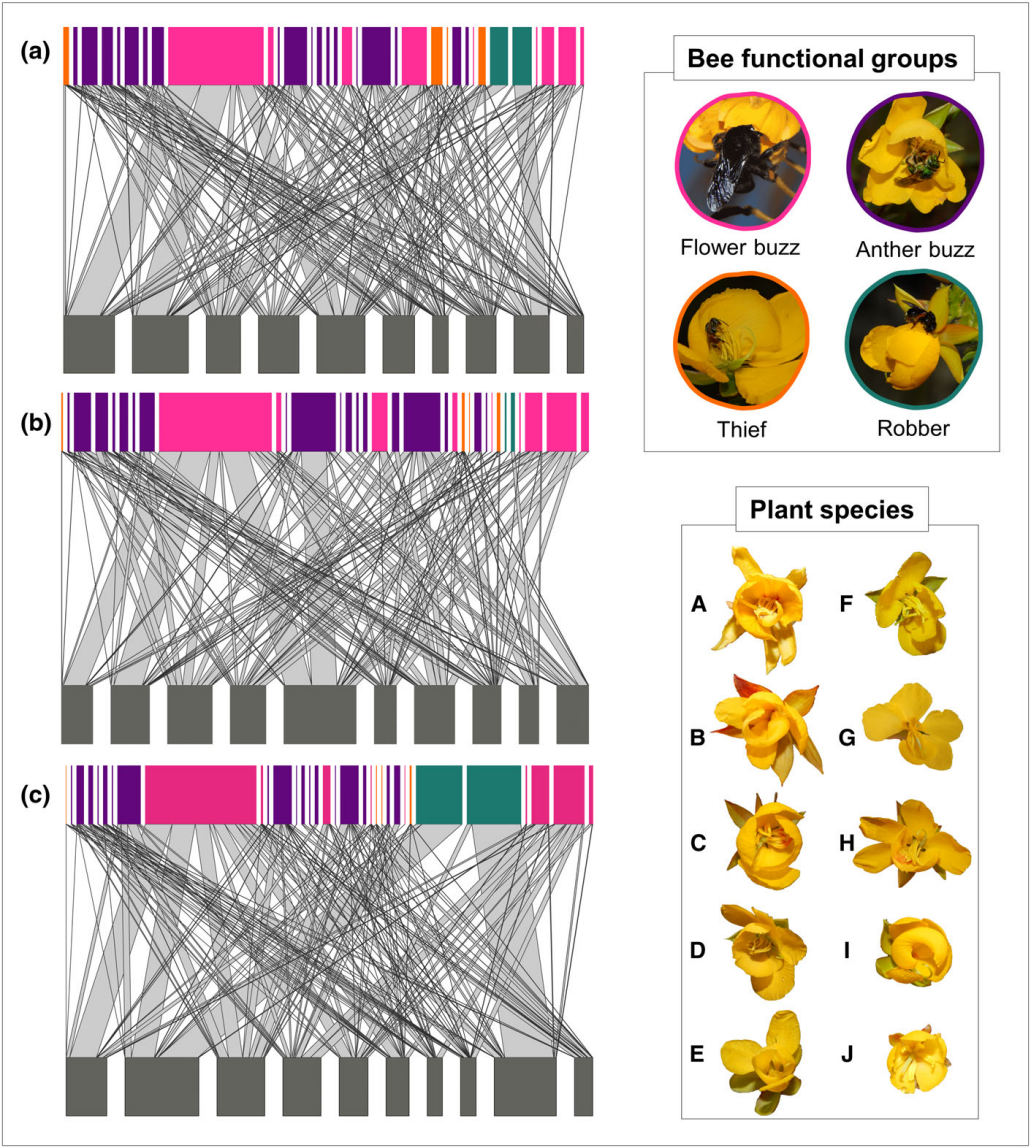
Bipartite networks illustrating the interactions among *Chamaecrista* species and bee visitors. (a) Frequency visitation network, (b) female performance network and (c) male performance network. Gray lines represent species interaction, and line thickness indicates the strength of interaction. The colours represent the functional groups for bees: flower buzzing (pink), anther buzzing (purple), theft (orange) and robbers (green). The plant species arranged in the network, from left to right, is the same sequence as the flower images (A–J). Key code: plant species (A) *Chamaecrista hedysaroides*, (B) *Chamaecrista bracteolata*, (C) *Chamaecrista nictitans*, (D) *Chamaecrista ramosa* var. 1, (E) *Chamaecrista debilis*, (F) *Chamaecrista desvauxii*, (G) *Chamaecrista flexuosa*, (H) *C. ramosa* var. 2, (I) *Chamaecrista distichoclada*, (J) *Chamaecrista rotundifolia*.

All types of networks (VN, FPN and MPN) were more specialised and modular than expected by the null models (Table 1; Supporting Information Fig. S1). For NODF, however, metrics were not significant (Table 1; Fig. S1). The MPN (visitation + pollen removal) network exhibited slightly higher specialisation and modularity than all other networks, whereas the FPN network (visitation + pollen deposition) showed lower values, but differences were at most 5% increase or decrease in metrics (Table 1; Figs 5, S1). Bee generality was relatively stable across the three networks and did not differ significantly among visitor groups. Plant generality varied more strongly between networks and was lower in the MPN (Table 1). Regarding species‐level specialisation, the VN, MPN and FPN did not differ significantly for plants or bees. This result was also observed concerning the strength of interactions between the VN and MPN for plants.

**Table 1:** Values of network metrics for visitation frequency, pollen deposition, pollen release and female and male performance networks.

Network			
Network metric	Visitation frequency network	Female performance network	Male performance network
Specialisation (*H*_2_′)	0.40	0.38	0.45*
Nestedness (*NODF*)	53.70	46.86	54.39
Modularity (*Q*_w_)	0.40	0.39	0.44*
Generality			
Plant	8.89	6.42	4.75*
Bees	4.78	4.76	4.58

* Significant differences (*P* ≤ 0.01) values obtained by the *r2dtable* null model.

The strength of interactions was higher in visitation frequency network than in FPN for bees (*t* = 3.271; *P* < 0.001) and for plants (*t* = 7.250; *P* < 0.001) as well as between female and MPNs for bees (*t* = −4.735; *P* < 0.001) and for plants (*t* = −7.250; *P* < 0.001) but was lower than MPN for bees (*t* = −4.852; *P* < 0.001). In the VN (Fig. S2a), most bee species (*c*. 70%) were classified as peripheral, exhibiting low *c* and *z* values (*z* ≤ 2.5 and *c* ≤ 0.62). This indicates that most bees only interacted with few species, primarily within their respective modules (i.e. peripheral roles). Additionally, 10 species were identified as module connectors (low *z* and high *c*). However, no species was classified as module or network hubs. Also, in the DN (Fig. S2b) and RN (Fig. S2c), no species was identified as module hubs or network hubs, with the majority classified as peripherals (60% and 67%, respectively) and 13 and 11 species identified as module connectors, respectively. The bee species *A. mellifera*, *Augochloropsis* sp. 50, *B. pauloensis*, *Caenohalictus* sp. 01, *C. tarsata*, *P. lineata*, *P. subnuda* and *X. hirsutissima* were consistently identified as module connector species across all three networks. Regarding plants, patterns were similar across networks, with most species classified as peripheral and no species classified as module or network hubs (Fig. S3a–c). Only *Chamaecrista hedysaroides* was consistently identified as a module connector species across all three networks. When assessing correlations among species‐level metrics, however, some divergences emerged.

While for the strength of bee species, we observed a positive correlation between VN and DN (pollen DN; cor = 0.72, *t* = 5.57, df = 29, *P* < 0.001), VN and pollen RN (cor = 0.76, *t* = 6.30, df = 29, *P* < 0.001) and between DN and RN (cor = 0.79, *t* = 6.91, df = 29, *P* < 0.001), for species complementary specialisation (*d*′), no correlation was observed among VN and DN or VN and RN (*P* > 0.05). Moreover, DN and RN showed a negative correlation (cor = −0.37, *t* = −2.18, df = 29, *P* < 0.05). For *c* values of bees in modular networks, we found positive correlations between all analysed network pairs: VN and DN (cor = 0.57, *t* = 3.69, df = 29, *P* < 0.001), VN and RN (cor = 0.62, *t* = 4.27, df = 29, *P* < 0.001) and DN and RN (cor = 0.72, *t* = 5.58, df = 29, *P* < 0.001). The same was found for *z* values: VN and DN (cor = 0.59, *t* = 3.90, df = 29, *P* < 0.001); VN and RN (cor = 0.61, *t* = 4.19, df = 29, *P* < 0.001) and DN and RN (cor = 0.68, *t* = 5.02, df = 29, *P* < 0.001). Finally, for plants, only species strength between DN and RN showed significant correlation (cor = 0.70, *t* = 2.7727, df = 8, *P* < 0.05).

## Discussion

Here we demonstrate that adding the quality components of plant–pollinator interactions to the pollination network affects our interpretation of network structure and species roles, as hypothesised. More specifically, plant male and female performances varied widely among pairwise interactions and when considering the bee functional groups. The predominant pollination partners were bees belonging to the *flower‐buzzing* functional group, as expected of a specialised pollen flower group such as *Chamaecrista* species. These differences in bee performance, while only translated into minor changes in network‐level metrics, did lead to greater differences when analysing species‐level metrics. Therefore, drawing ecological and evolutionary conclusions solely from visitation data may be misleading in the context of species roles in pollination network analyses and their ecological and evolutionary consequences.

### More than visits: morphology and behaviour shape pollinator contributions to effective pollination

Bee visitors to *Chamaecrista* were highly dissimilar considering the frequency of visitation, and the capacity to deposit and remove pollen, as reported for other studies that evaluated pollination of different plant species with buzz‐pollinated flowers (Mesquita‐Neto *et al*., 2018, 2021; Barbosa *et al*., 2023). Nonetheless, differences in species composition and ecological conditions may influence observed patterns. Species from the *flower‐buzzing* group, such as *Bombus* and *Xylocopa* species, are large, spend less time at each flower, manipulate the anthers and make shorter vibrational pulses (see also Mesquita‐Neto *et al*., 2018, 2021). These bee species are capable of effectively adjusting pollen‐collection behaviour to quickly extract large amounts of pollen from poricidal anthers (Buchmann, 1983; De Luca *et al*., 2013; Burkart *et al*., 2014; Russell *et al*., 2017; Mesquita‐Neto *et al*., 2021). Besides that, by vibrating the flowers, they touch the stigma on most visits, as their body size usually exceeds the gap between the anthers and the stigma (i.e. herkogamy), ensuring contact with both reproductive structures simultaneously (Solís‐Montero & Vallejo‐Marín, 2017; Barbosa *et al*., 2023). Considering that the vibratory behaviour of bees is a specialised function (Vallejo‐Marín, 2019), larger bees that vibrate flowers while embracing all or most of the anthers are more likely to promote pollination. Nevertheless, these bees are the main contributors to hetero‐specific pollen deposition in pollen flower communities with high floral diversity (Mesquita‐Neto *et al*., 2018). By contrast, the *anther‐buzzing* bees, which are typically smaller, produce longer vibrational pulses on anthers separately each time, overall spend more time on flowers (Mesquita‐Neto *et al*., 2018; Barbosa *et al*., 2023), and remove and deposit less pollen. Nevertheless, some of these smaller bees show behaviours that could compensate for the poor fit with flowers to achieve levels of pollen release and deposition close to larger flower‐buzzing bees (De Luca *et al*., 2013; Rosi‐Denadai *et al*., 2020; Barbosa *et al*., 2023). For instance, smaller bees such as *Augochloropsis* and *Melipona* species spend more time manipulating the anthers and change their position during the visit (see Mesquita‐Neto *et al*., 2021). These behavioural traits help explain why anther‐vibrating bees, despite their smaller size and less precise fit with floral morphology, are generally more effective than nonvibrating species and can function as secondary pollinators.

Nonvibratory bees are commonly described as antagonists in buzz pollination systems by often damaging floral structures (De Luca & Vallejo‐Marín, 2013; Mesquita‐Neto *et al*., 2021). Particularly, species from the *robber* group always exhibited the behaviour of cutting the petals, decreasing the visual attractiveness of flowers (see Hargreaves *et al*., 2009; Rego *et al*., 2018), as well as chewing the anthers to collect pollen. So, this destructive behaviour of anthers leads to the loss of available floral resources (i.e. pollen grain) for more effective pollinators, impacting male fitness (Mesquita‐Neto *et al*., 2018, 2021). Moreover, the observed damage of the modified petal, known as the *cucullus*, results in the loss of the specialised pollination mechanisms mediated by floral traits in the buzz pollination (Monteiro *et al*., 2025). The other group of nonvibrating bees included those classified as *thieves*. Although they did not damage floral structures and collected pollen from apical pores or the remaining pollen onto petals, they were the least efficient at removing pollen and rarely deposited it onto stigmas. So, while they may cause some negative effects by acting as potential competitors for resources, their effect on the reproductive success of plant species must be low.

### Pollination networks underestimate male function when based solely on visitation

Previous studies suggested that networks incorporating effectiveness components are more specialised than the ones considering only visitation frequency, as not all floral visitors are truly effective pollinators even when contacting the reproductive structures (King *et al*., 2013; Ballantyne *et al*., 2015; Santiago‐Hernández *et al*., 2019). However, our results contradict these findings, as networks constructed from female components diverged little on overall network metrics and were even slightly more generalist than the one based solely on visitation. Such small differences may be explained by a minor variation in pollen deposition among most bee–flower combinations within this specialised pollination system in *Chamaecrista*, where 80% of the recorded bees exhibited vibratory behaviour during flower manipulation. Even though flowers in the genus (including the ones studied here) show considerable variation in size and the morphology of the petals and anthers, there is some conformity (Gottsberger & Silberbauer‐Gottsberger, 1988; Arceo‐Gómez *et al*., 2011; Almeida *et al*., 2013a,b; Nogueira *et al*., 2018), and adequate fit with most buzzing bees, implying strong selective pressures to optimise pollination (Solís‐Montero & Vallejo‐Marín, 2017; Mesquita‐Neto *et al*., 2021). Most bee species positioned themselves enclosed in the flowers during anther vibration, providing the necessary morphological matching to ensure contact with the reproductive structures of the flowers (Valadão‐Mendes *et al*., 2022; Vallejo‐Marín, 2022; Barbosa *et al*., 2023).

By contrast, the male components of pollination (i.e. pollen release) conformed with the expectation that incorporating qualitative measures increases specialisation of networks, reflecting a wider variation of bees' species in performance (King *et al*., 2013; Ballantyne *et al*., 2015; Mesquita‐Neto *et al*., 2018; Santiago‐Hernández *et al*., 2019; Pearson *et al*., 2023; Weinman *et al*., 2023). In systems involving pollen‐only flowers, plants confront the challenge of mitigating excessive pollen loss while ensuring continued attractiveness to pollinators (Opedal *et al*., 2023). Consequently, restricting pollen release through poricidal anthers (Buchmann, 1983), which can only be released by bees capable of performing specific vibrations (Vallejo‐Marín, 2022), may lead to higher variation in the male performance compared with the female one, and consequent higher specialisation of this network.

Our results indicate that, depending on the type of data used, VNs may somehow underestimate the degree of specialisation between plants and pollinators, particularly by failing to capture the contribution of male reproductive performance. Despite this, the higher consistency of bee species level indices across network currencies, as suggested by positive and significant correlations, and lower variation in generality for this group, suggest that bee foraging breadth and network roles are relatively robust to how interactions are quantified, whereas for plants methodological choices are more relevant. Such inaccurate estimates may be stronger when considering species‐level metrics, which were often uncorrelated, especially for plants. Therefore, while estimating pollinator species performance through network approaches may be more reliable (Vázquez *et al*., 2012), it may not be the case when assessing the plant side of the interaction (Santiago‐Hernández *et al*., 2019). Our results reinforce that analysing plant–pollinator interactions based only on visitation frequency may not provide an accurate understanding of the pollinator's importance in pollination systems. Thus, the incorporation of data on pollinator effectiveness into network construction will contribute to furthering our understanding of pollination networks.

### Conclusion

Considering the multifaceted role of bee pollination effectiveness within *Chamaecrista* species and the diverse functional groups of bees involved in these pollination systems, our comprehensive analysis highlights the nuanced impact of various bee species on plant reproductive success. Specifically, the assessment of pollination effectiveness by a floral visitor is essential to comprehend its impact on the reproductive success of plants. Obtaining a precise measure of this effectiveness is crucial for understanding the ecology and evolution of interactions between plants and pollinators. By examining the variation in pollination effectiveness, it is possible to identify which floral visitors play the main role as pollinators, whereas others provide minimal benefits or act as thieves. This understanding is crucial for determining which pollinators genuinely contribute to the fitness of plants, including both male and female components, thereby enhancing the reliability and predictive power of network analyses in studies of plant–pollinator interactions. Therefore, we suggest that future studies consider integrating distinct qualitative measures of interactions, along with quantitative measures, to assess the structure and functioning of pollination systems characterised as ecological networks. Furthermore, this information can provide valuable insights into the intensity of selective pressures exerted by each floral visitor, fundamental for understanding the evolutionary aspects of specialised pollination systems, such as buzz pollination.

## Competing interests

None declared.

## Author contributions

LBV‐M, PKM and VLGB conceived and designed the research, with input from all co‐authors on the study's conceptual development. LBV‐M collected and processed all field data. LBV‐M and PCS performed the analyses and prepared the figures and tables. LBV‐M led the writing, with PKM, VLGB, PCS and ARR contributing substantially to improve the manuscript. All authors approved the final version.

## Disclaimer

The New Phytologist Foundation remains neutral with regard to jurisdictional claims in maps and in any institutional affiliations.

## Supporting information


**Fig. S1** Boxplots of network metrics for each network type.
**Fig. S2** Bee species categorised by within‐ and among‐module connectivity (*c* and *z* values) across networks.
**Fig. S3** Plant species categorised by within‐ and among‐module connectivity (*c* and *z* values) across networks.
Please note: Wiley is not responsible for the content or functionality of any Supporting Information supplied by the authors. Any queries (other than missing material) should be directed to the *New Phytologist* Central Office.

## Data Availability

Related codes are available on GitHub (https://github.com/SantanaPC/NetworkCurrencies).
