# Supplementary material for: Fine‐tuning the buzz: comparing visitation frequency and pollination effectiveness in plant–pollinator networks
Source: New Phytol. 2025 Nov 21;249(4):2140–52. doi: 10.1111/nph.70758 (PMC12825406; doi:10.1111/nph.70758)

New Phytologist

New Phytologist Supporting Information

Article title: Fine-tuning the buzz: comparing visitation frequency and pollination effectiveness in plant-pollinator networks

Authors: Lorena B. Valadão-Mendes, Pamela C. Santana, André R. Rech, Vinícius L. G. Brito, Pietro K. Maruyama

Article acceptance: 28 October 2025

Fig. S1 Box plots of metrics of each network. (a) Level of specialisation (H_2') (b) Level of nestedness (*NODF*) (c) Level of modularity (Q_w) (d) Generality of plant and bee species. Key code: (VN) Frequency visitation network. (DN) Pollen deposition network (RN) Pollen release network (FPN) Female performance network (MPN) Male performance network.

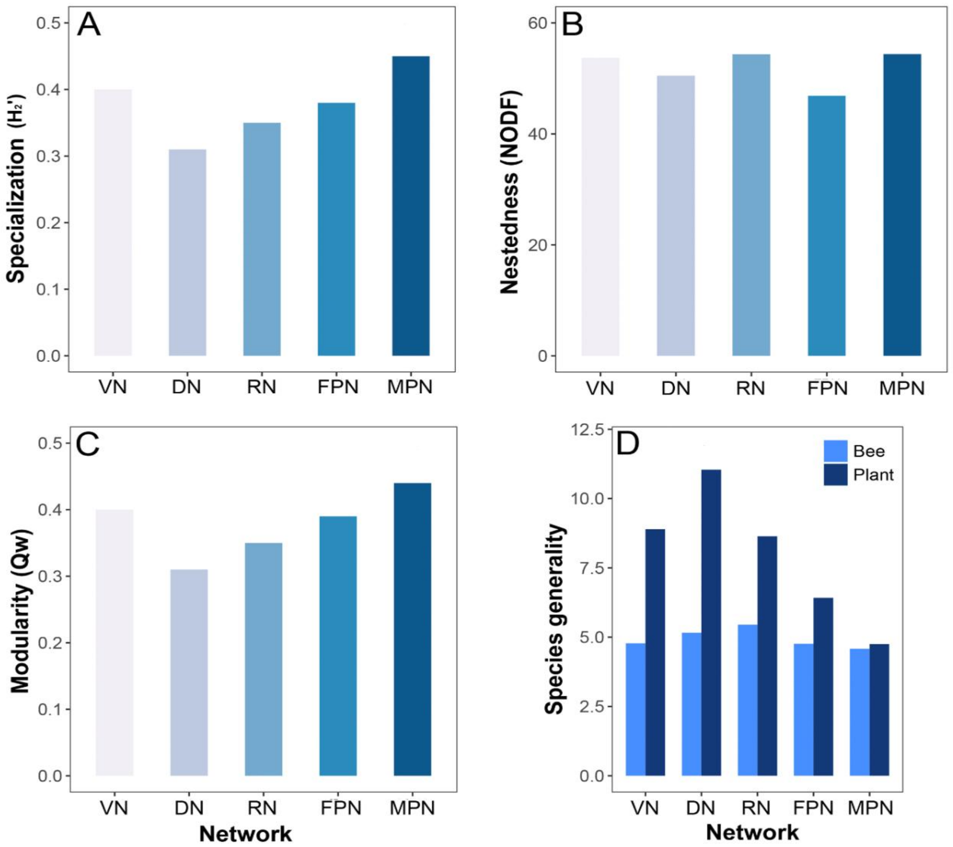

Fig. S2 Bee species categorized based on their connectivity within and between modules (c and z-values). (a) Frequency visitation network (b) Pollen deposition network (c) Pollen release network. The colours represent the bee functional group: flower buzzing (pink), anther buzzing (purple), thief (orange), robbers (green).

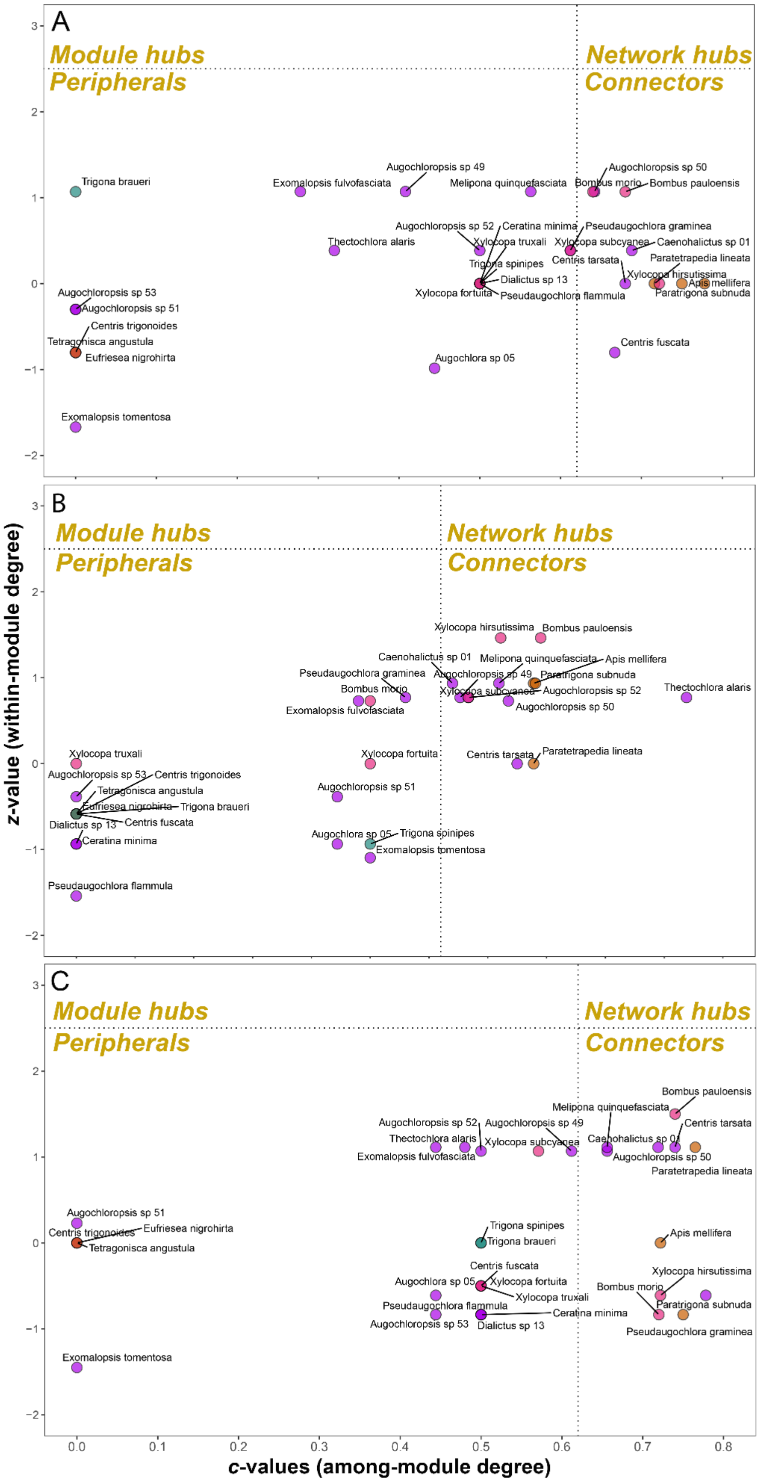

Fig. S3 Plant species categorized based on their connectivity within and between modules (c and z-values). (a) Frequency visitation network (b) Pollen deposition network (c) Pollen release network.

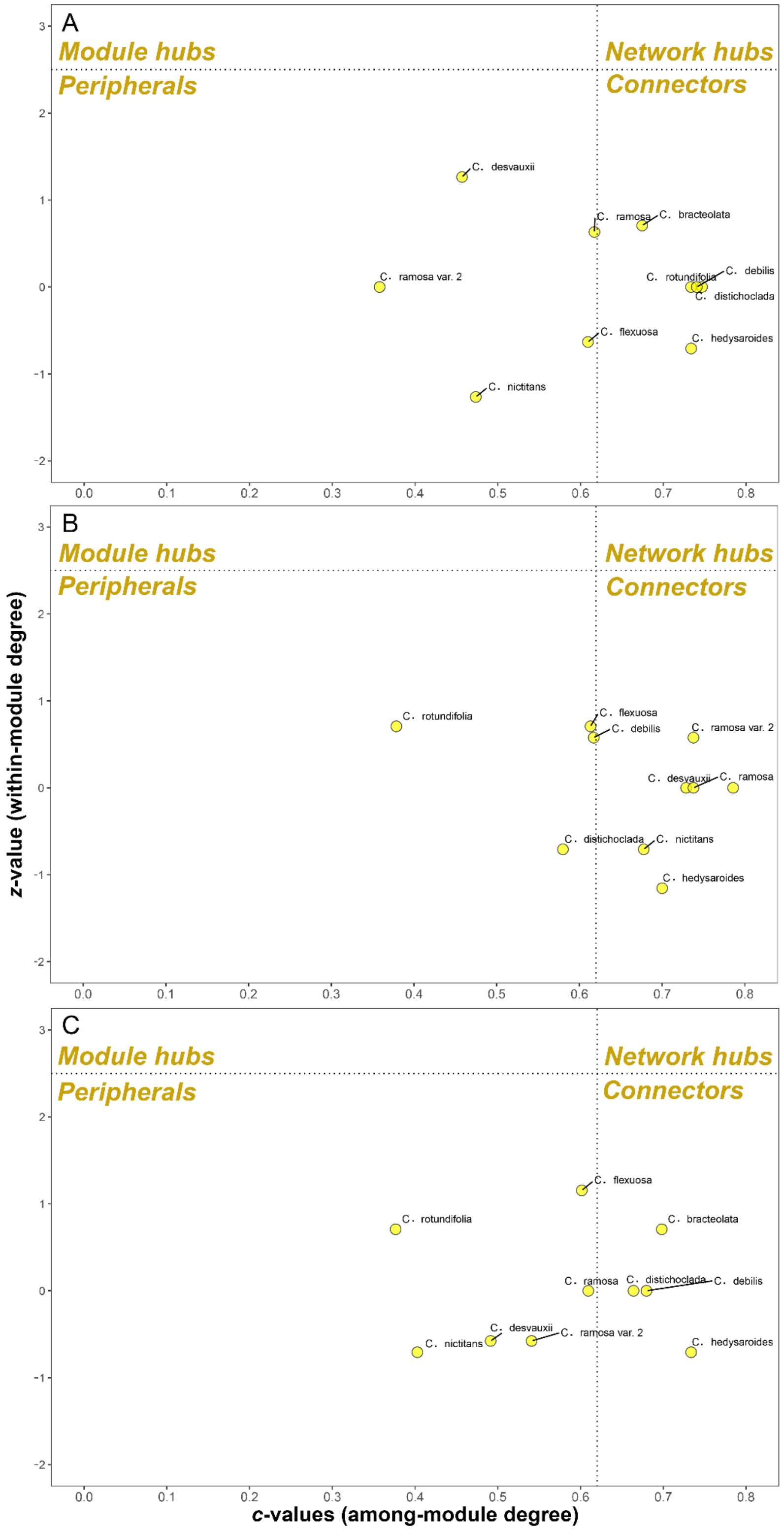

Supplement: Supplementary file 1 — Fig. S1 Boxplots of network metrics for each network type. Fig. S2 Bee species categorised by within‐ and among‐module connectivity (c and z values) across networks. Fig. S3 Plant species categorised by within‐ and among‐module connectivity (c and z values) across networks. Please note: Wiley is not responsible for the content or functionality of any Supporting Information supplied by the authors. Any queries (other than missing material) should be directed to the New Phytologist Central Office. [file NPH-249-2140-s001.pdf]
